# From depigmentation to dysmetabolism: epicardial fat thickness and atherogenic lipid ındices in vitiligo^؆^

**DOI:** 10.1016/j.abd.2026.501428

**Published:** 2026-08-17

**Authors:** Mustafa Esen, Abdullah Demirbas, Esin Diremsizoglu, Murat Harman, Kamran İldırımlı

**Affiliations:** a Department of Dermatology, Faculty of Medicine, Dicle University, Diyarbakır, Turkey; b Department of Dermatology, Faculty of Medicine, Kocaeli University, Kocaeli, Turkey; c Department of Cardiology, Faculty of Medicine, Fırat University, Elazıg, Turkey; d Department of Cardiology, Faculty of Medicine, Dicle University Diyarbakır, Turkey

**Keywords:** Adipose tissue, Atherosclerosis, Cardiometabolic risk factors, Echocardiography, Inflammation, Oxidative stress, Vitiligo

## Abstract

**Background:**

Systemic metabolic and cardiovascular alterations have been reported in vitiligo, yet Epicardial Fat Thickness (EFT) and echocardiographic structural changes remain understudied. This study compares these parameters between vitiligo patients and healthy controls and examines their association with disease severity.

**Methods:**

In this case–control study, 90 adults with vitiligo and 90 matched controls underwent standardized transthoracic echocardiography with multi-window EFT assessment (parasternal, apical, subcostal). Clinical and lipid indices were recorded. Associations with disease severity were evaluated using Spearman correlations and multivariable linear regression, adjusting for age, sex, BMI, blood pressure, LDL, fibrinogen and CRP. ROC analyses assessed the discriminatory value of biomarkers for high-severity disease.

**Results:**

Vitiligo patients demonstrated significantly higher EFT across all echocardiographic windows (p ≤ 0.002) and larger left atrial and ascending aorta diameters (p ≤ 0.010). In multivariable models, vitiligo independently predicted mean EFT (B = 0.82, p = 0.003) and aortic valve gradient (B = 1.13, p = 0.019). VASI/BSA correlated strongly with triglycerides, VLDL, TG/HDL ratio, and atherogenic index of plasma (*r* = 0.574–0.681, all p < 0.001), and with both aortic gradient and EFT. The acrofacial phenotype exhibited the most adverse metabolic and echocardiographic profiles.

**Study limitations:**

Comorbidity-based exclusion criteria enhance internal validity but limit generalizability, and systematic lifestyle data were unavailable.

**Conclusions:**

Vitiligo is independently associated with increased epicardial fat, adverse triglyceride-related atherogenic indices, and subtle cardiac structural alterations, even without overt comorbidities. These findings are most pronounced in patients with higher VASI severity and acrofacial or widely distributed disease, supporting consideration of cardiovascular assessment in these subgroups.

## Introduction

Vitiligo is a chronic, immune-mediated disorder characterized by selective melanocyte destruction driven by oxidative stress, innate immune activation, and interferon-gamma (IFN-γ)–mediated chemokine signaling.^1^, ^2^, ^3^ Although historically considered a skin-limited condition, growing evidence indicates that vitiligo is accompanied by systemic immune activation, low-grade inflammation, and metabolic disturbances extending beyond the epidermis.^4^, ^5^ These immunometabolic abnormalities overlap with pathways involved in endothelial dysfunction, insulin resistance, and atherosclerosis, prompting increasing interest in the cardiovascular health of patients with vitiligo.^6^ Recent epidemiologic data suggest that individuals with vitiligo may have higher rates of metabolic syndrome, dyslipidemia, hypertension, and cardiovascular events compared with the general population, supporting the view of vitiligo as a systemic inflammatory condition in which cardiometabolic assessment may be clinically relevant.^7^, ^8^

Similar associations have been demonstrated in other chronic inflammatory dermatoses, including psoriasis, hidradenitis suppurativa, lichen planus, rosacea, seborrheic dermatitis, and androgenetic alopecia, where increased epicardial fat tissue (EFT) and adverse lipid profiles have been consistently reported.^9^, ^10^, ^11^, ^12^, ^13^, ^14^, ^15^ EFT is a metabolically active visceral fat depot adjacent to the myocardium and coronary arteries, capable of secreting proinflammatory cytokines such as interleukin-6 (IL-6) and tumor necrosis factor-alpha (TNF-α), and is considered a noninvasive marker of systemic inflammation and cardiometabolic risk, independent of body mass index.^16^, ^17^ Despite these insights, EFT and broader echocardiographic findings have not been adequately investigated in vitiligo, and the cardiometabolic profile of this population remains insufficiently defined.

This study, therefore, aimed to evaluate echocardiographic, inflammatory, and lipid-based cardiometabolic parameters in patients with vitiligo compared with matched controls, and to assess whether these indices vary with disease severity and clinical phenotype to help identify subgroups with potentially higher cardiometabolic risk.

## Methods

This prospective case–control study included 90 patients with vitiligo and 90 age- and sex-matched healthy controls recruited from the dermatology and cardiology outpatient clinics between September 2023 and May 2024. The protocol was approved by the local ethics committee (Approval nº 2023/09-30), and written informed consent was obtained from all participants in accordance with the Declaration of Helsinki.

Participants were excluded if they had a history of cardiovascular disease (coronary artery disease, heart failure, arrhythmia, or cardiomyopathy), hypertension, diabetes mellitus, dyslipidemia, thyroid disorders (absence of a prior diagnosis), chronic renal or hepatic impairment, rheumatologic or other chronic inflammatory diseases, cerebrovascular or peripheral vascular disease, active infection, pregnancy, or regular alcohol consumption. Individuals receiving systemic corticosteroids, immunosuppressants, biologic agents, lipid-lowering, or antihypertensive therapy were excluded. Controls were recruited among hospital staff and individuals attending for non-inflammatory dermatological complaints, and only those without systemic disease or regular medication use were included.

For all participants, demographic data and clinical history were recorded. In the vitiligo group, disease duration, subtype (focal, acrofacial, segmental, generalized), family history, and activity status were documented. Disease activity was defined as the presence of new lesions or the Koebner phenomenon within the preceding three months. Disease extent was quantified using body surface area (BSA) involvement based on the rule of nines, and disease severity was assessed with the Vitiligo Area Scoring Index (VASI) using standardized body diagrams.^18^ VASI scores were subsequently categorized into low, moderate, and high severity groups according to tertile-based distributions.

All participants underwent standardized anthropometric measurements, including height, weight, and body mass ındex (BMI), obtained using calibrated devices. Systolic and diastolic blood pressures were measured after ten minutes of rest in the seated position, and the mean of two readings was recorded. After a minimum 12-h overnight fast, venous blood samples were obtained to measure complete blood count parameters and biochemical variables(fasting glucose, urea, creatinine, albumin, total cholesterol, HDL-C, LDL-C, and triglycerides) using automated analyzers in the same accredited laboratory. C-reactive protein (CRP) was measured by immunoturbidimetric assay. Derived indices were calculated for each participant, including very-low-density lipoprotein (VLDL = triglycerides/5), Triglyceride-to-HDL ratio (TG/HDL), and the Atherogenic Index of Plasma (AIP = log_10_[TG/HDL]).

Transthoracic echocardiography was performed in all subjects in the left lateral decubitus position using a Philips EPIQ-7 ultrasound system with a 7 to 2 MHz transducer. Echocardiographic examinations were performed by experienced cardiologists, who were blinded to clinical, laboratory, and dermatological data throughout the study. Imaging modalities included M-mode, two-dimensional, and Doppler (pulsed, continuous-wave, and color) techniques. Left ventricular end-diastolic and end-systolic diameters, left atrial and right ventricular diameters, interventricular septal thickness, aortic root and ascending aorta diameters, and left ventricular (LV) ejection fraction (Simpson’s biplane method) were measured. Aortic valve gradient was assessed by continuous-wave Doppler. Each parameter was averaged over three consecutive cardiac cycles.

EFT was defined as the echo-free space between the visceral pericardium and the outer myocardial wall of the right ventricle. EFT was measured at end-diastole from the parasternal long-axis, apical four-chamber, and subcostal views. For each window, measurements were obtained over three cardiac cycles and averaged; the overall mean EFT was calculated as the average of the three view-specific means.

### Statistical analysis

Statistical analyses were performed using SPSS v 29 (IBM Corp., Armonk, NY, USA). Continuous variables were assessed for normality with the Kolmogorov-Smirnov test. Between-group comparisons (vitiligo vs controls) were conducted using the Student’s *t*-test for normally distributed data and the Mann-Whitney *U*-test for non-normally distributed data. Categorical variables were compared with the Chi-Square or Fisher’s exact test, as appropriate. Standardized mean differences (SMDs) were calculated to quantify the magnitude of between-group differences in baseline characteristics. Correlations between vitiligo severity measures (VASI and BSA) and cardiometabolic or echocardiographic parameters were examined using Spearman’s correlation coefficients. Multivariable linear regression analyses were used to identify independent determinants of selected echocardiographic parameters (including EFT), adjusting for age, sex, BMI, systolic and diastolic blood pressure, LDL-C, fibrinogen and CRP. Receiver Operating Characteristic (ROC) curves were generated to evaluate the discriminatory performance of lipid indices for identifying patients with high VASI severity. A two-tailed p-value < 0.05 was considered statistically significant.

## Results

### Baseline clinical and echocardiographic characteristics

Ninety patients with vitiligo and 90 matched controls were included. Age, sex, and BMI were comparable between groups. Vitiligo patients had higher diastolic blood pressure (76.5 vs. 70 mmHg, p = 0.040), CRP (2.50 vs. 1.49 mg/L, p = 0.006), and fasting glucose (96 vs. 93 mg/dL, p = 0.012). Echocardiographically, the vitiligo group showed a larger left atrial diameter (33 vs. 31 mm, p = 0.002) and ascending aorta diameter (31 vs. 30 mm, p = 0.010), with a higher aortic valve gradient (6 vs. 4.5 mmHg, p = 0.016). Epicardial fat thickness was higher across parasternal long-axis (p < 0.001), apical four-chamber (p = 0.001), and subcostal views (p = 0.002). LV end-diastolic and end-systolic diameters were smaller in the vitiligo group (p = 0.006 and p < 0.001), while ejection fraction did not differ (p = 0.562). Although these differences were statistically significant, all echocardiographic measurements in both groups remained within normal reference ranges (Table 1).

**Table 1:** Baseline demographic, laboratory, and echocardiographic characteristics of vitiligo patients and controls, with Standardized Mean Differences (SMD).

Variable	Control (n = 90)	Vitiligo (n = 90)	p-value	Effect Size (*r*)	SMD
**Age (years)**	37 (25.8–47.0)	38.5 (26.0–49.0)	0.402	*r* = 0.065	0.159
**Sex Male n (%)**	46 (51.1%)	58 (64.4%)	0.070	RR = 1.26	0.267
**Female n (%)**	44 (48.9%)	32 (35.6%)			
**BMI (kg/m^2^)**	25.69 (22.49–29.29)	23.88 (22.22–27.14)	0.268	*r* = 0.086	-0.145
**Systolic BP (mmHg)**	120 (110–130)	120 (112–124)	0.114	*r* = 0.123	0.338
**Diastolic BP (mmHg)**	70 (63.8–80)	76.5 (70–80)	**0.040**	*r* = 0.161	0.345
**Fasting Blood Glucose (mg/dL)**	93 (89–100)	96 (90–104.5)	**0.012**	*r* = 0.195	0.260
**Urea (mg/dL)**	29.2 (24.6–36.0)	28.6 (24–34)	0.754	*r* = 0.024	0.042
**Creatinine (mg/dL)**	0.745 (0.62–0.87)	0.75 (0.70–0.91)	0.053	*r* = 0.151	**0.310**
**Albumin (g/L)**	45.06 (42–47.16)	46.60 (42.87–47.75)	0.173	*r* = 0.106	0.165
**CRP (mg/L)**	1.49 (0.62–3.16)	2.50 (1.12–5.00)	**0.006**	*r* = 0.216	0.430
**Total Cholesterol (mg/dL)**	176 (153–217)	195.5 (162–221)	0.511	*r* = 0.051	-0.041
**Non-HDL (mg/dL)**	129.1 (100.7–167.2)	141.5 (117–170)	0.252	*r* = 0.089	0.105
**VLDL (mg/dL)**	24.0 (17.0–40.0)	22.0 (13.8–40.0)	0.302	*r* = 0.078	0.003
**Triglycerides (mg/dL)**	120 (85–200)	111.5 (69–200)	0.302	*r* = 0.081	**0.003**
**HDL (mg/dL)**	49 (42.2–63)	46 (39–54)	0.088	*r* = 0.133	-0.363
**LDL (mg/dL)**	111 (88–141)	114.3 (92.4–132.8)	0.900	*r* = 0.010	-0.155
**TG/HDL ratio**	2.53 (1.43–3.95)	2.49 (1.46–4.17)	0.922	*r* = 0.007	0.072
**AIP**	0.402 (0.156–0.597)	0.397 (0.165–0.620)	0.922	*r* = 0.007	0.046
**Fibrinogen (mg/dL)**	283 (240–329)	285.5 (234–334)	0.739	*r* = 0.025	-0.050
**Aortic Root (mm)**	30 (28–33)	30 (28–33)	0.650	*r* = 0.034	-0.136
**Left Atrium (mm)**	31 (30–33)	33 (31–35)	**0.002**	*r* = 0.228	0.453
**Ascending Aorta (mm)**	30 (28–31)	31 (30–34)	**0.010**	*r* = 0.193	0.348
**Right Ventricle (mm)**	32 (30–38)	32 (30–33)	**0.017**	*r* = 0.178	-0.353
**LV Diastole (mm)**	46 (42–49)	43 (41–45)	**0.006**	*r* = 0.204	-0.371
**LV Systole (mm)**	35 (30–38)	31 (30–33)	**<0.001**	*r* = 0.314	-0.723
**IVS (mm)**	10 (8–10)	10 (8–10)	**0.021**	*r* = 0.172	0.268
**Aortic Gradient (mmHg)**	4.5 (2–8)	6 (5–7.25)	**0.016**	*r* = 0.179	0.169
**Ejection Fraction (%)**	60 (60–60)	60 (60–60)	0.562	*r* = 0.043	0.086
**EFT PLAX (mm)**	2 (1–4)	4 (2–4)	**<0.001**	*r* = 0.302	0.539
**EFT A4C (mm)**	2 (1–4)	3 (2–4.25)	**0.001**	*r* = 0.255	0.308
**EFT Subcostal (mm)**	2 (1–4)	3 (2–4)	**0.002**	*r* = 0.248	0.294
**EFT Mean (mm)**	2 (1.3–4)	3.65 (2–4.18)	**0.001**	*r* = 0.270	0.390
**Vitiligo Type**					
Focal		39 (43.3%)			
Acrofacial		25 (27.8%)			
Segmental		15 (16.7%)			
Generalized		11 (12.2%)			
**Activity Status**					
Stable		79 (87.8%)			
Active		11 (12.2%)			
**Vitiligo duration (months)**		73 (24–153)			
**Family history (yes)**		10 (11.1%)			
**BSA (%)**		5 (5–10)			
**VASI**		7.31 (4.72–12.65)			

Continuous variables are presented as Median (IQR) and compared using the Mann-Whitney *U*-test; effect size is reported as *r*. Categorical variables are expressed as n (%). Vitiligo-specific clinical features are shown only for the vitiligo group.

BMI, Body Mass Index; BP, Blood Pressure; CRP, C-Reactive Protein; HDL, High-Density Lipoprotein; LDL, Low-Density Lipoprotein; Non-HDL, Total cholesterol minus HDL; TG/HDL, Triglyceride-to-HDL ratio; AIP, Atherogenic Index of Plasma; IVS, Interventricular Septum; EF, Ejection Fraction; EFT, Epicardial Fat Thickness; PLAX, Parasternal Long Axis; A4C, Apical four-Chamber view. Standardized Mean Differences (SMDs) were calculated to quantify the magnitude of between-group differences independently of sample size. Bold values indicate statistically significant p-values (p < 0.05).

### Correlations of vitiligo severity with cardiac structure and ınflammation

**Figure 1:**
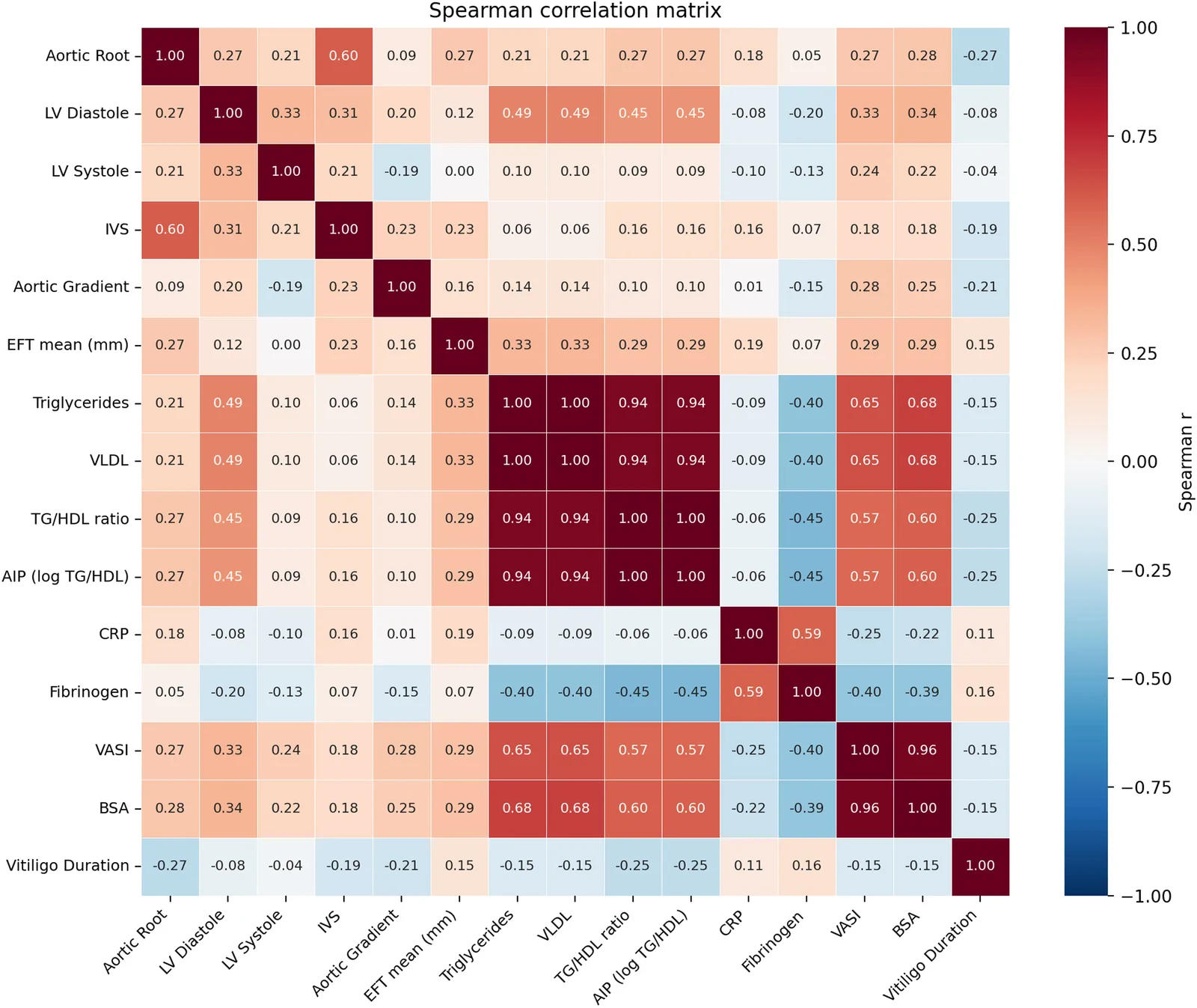
**Correlation heatmap of echocardiographic, inflammatory, and cardiometabolic parameters in patients with vitiligo.** The heatmap illustrates pairwise Spearman correlation coefficients among vitiligo duration, vitiligo severity measures (VASI, BSA), echocardiographic indices (aortic root, LV diameters, IVS, aortic gradient, mean EFT), cardiometabolic markers (triglycerides, VLDL, TG/HDL ratio, AIP), and inflammatory markers (CRP, fibrinogen).

Higher vitiligo severity (VASI, BSA) was associated with adverse cardiometabolic and structural patterns. Both VASI and BSA correlated positively with aortic root (*r* = 0.269–0.277; p = 0.010–0.008), LV diastolic diameter (*r* = 0.335–0.343; p = 0.001), LV systolic diameter (*r* = 0.219–0.244; p = 0.039–0.021), aortic gradient (*r* = 0.246–0.276; p = 0.019–0.009), and mean EFT (*r* = 0.292–0.294; p = 0.005). Triglyceride-related markers showed the strongest associations: triglycerides/VLDL (*r* = 0.647–0.681; p < 0.001) and TG/HDL ratio/atherogenic index of plasma (AIP; *r* = 0.574–0.601; p < 0.001). Fibrinogen showed an inverse association with severity (*r* = -0.394 to -0.395; p < 0.001). Mean EFT additionally correlated with left atrial diameter (*r* = 0.307, p = 0.003) and intraventricular septum thickness (IVS; *r* = 0.227, p = 0.031). Vitiligo duration correlated negatively with aortic root (*r* = -0.267, p = 0.011) and TG/HDL ratio/AIP (*r* = -0.251, p = 0.020), but showed no significant association with mean EFT or triglycerides/VLDL (all p ≥ 0.166) (Fig. 1).

### Independent determinants of echocardiographic measurements in vitiligo

**Figure 2:**
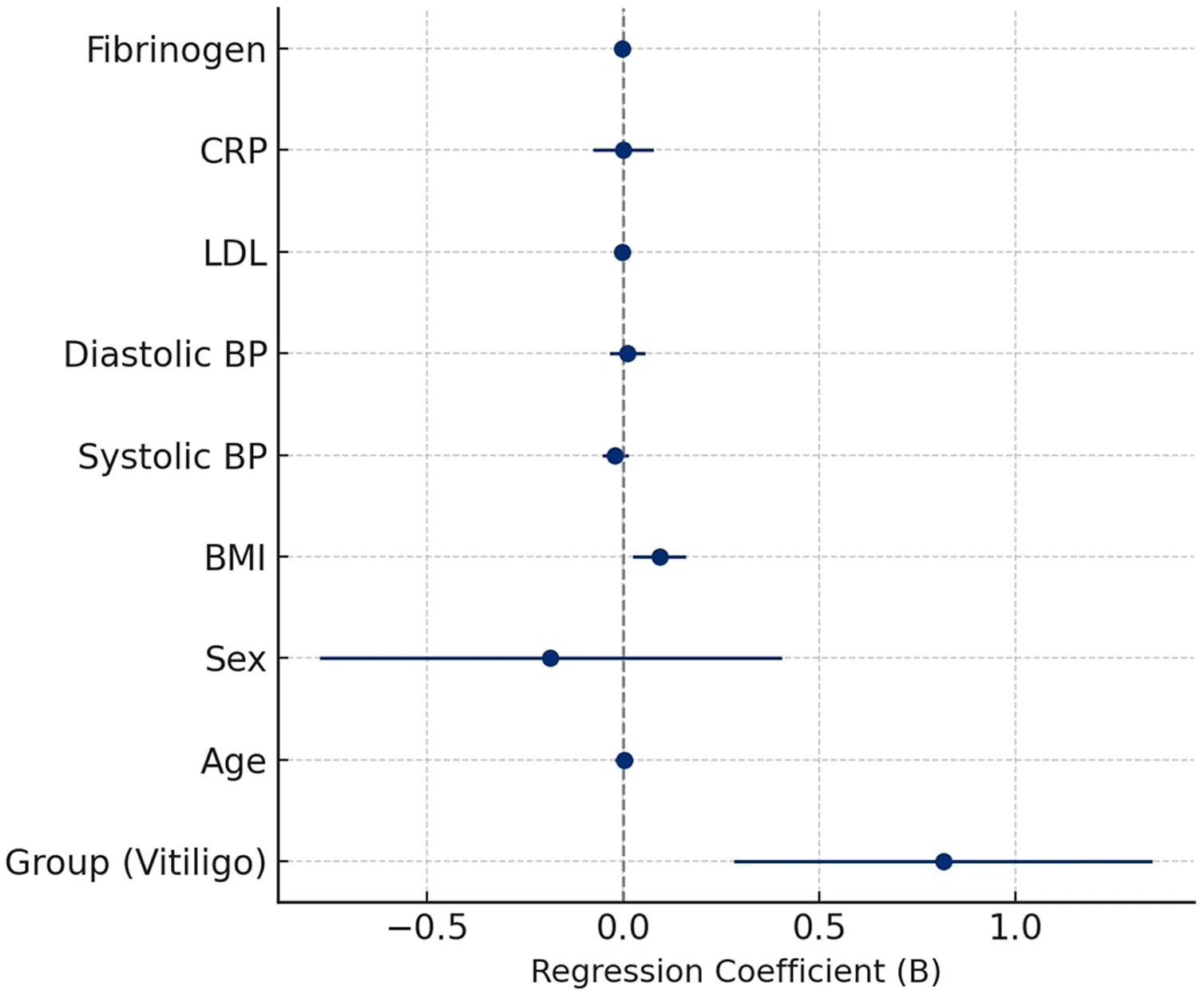
**Forest plot showing multivariable regression coefficients (B) for determinants of mean epicardial fat thickness.** The model adjusts for age, sex, BMI, systolic and diastolic blood pressure, LDL, CRP, and fibrinogen. Vitiligo diagnosis was an independent predictor of increased EFT.

In multivariable models adjusted for demographic, metabolic, and inflammatory covariates, vitiligo remained an independent predictor of increased EFT across all echocardiographic windows, including mean EFT (B = 0.82, p = 0.003), parasternal (B = 1.09, p < 0.001), apical (B = 0.73, p = 0.013), and subcostal measurements (B = 0.67, p = 0.012) (Fig. 2). Vitiligo was also independently associated with a higher aortic valve gradient (B = 1.13, p = 0.019), a larger left atrial diameter (B = 1.20, p = 0.012), and a smaller right ventricular diameter (B = -1.83, p = 0.005).

Age, male sex, and BMI were consistent determinants of left atrial and aortic dimensions, interventricular septal thickness, and diastolic function; age independently predicted left atrial diameter and aortic gradient (both p < 0.001), while male sex was inversely associated with left atrial diameter and IVS thickness (both p < 0.001).

### Subgroup differences according to vitiligo severity, activity, subtype, and BSA involvement

Across VASI tertiles, the high VASI group exhibited a more adverse cardiometabolic profile, with significantly higher triglycerides (p < 0.001), VLDL (p < 0.001), TG/HDL ratio (p < 0.001), and AIP (p < 0.001). Echocardiographically, these patients demonstrated a larger aortic root diameter (p = 0.003), a higher aortic valve gradient (p = 0.028), and higher epicardial fat thickness across parasternal, apical, and subcostal views (p = 0.045, 0.042, and 0.002, respectively) (Table 2). Right ventricular diameter also differed modestly across tertiles (p = 0.022), whereas other structural parameters were not significantly different.

**Table 2:** Inflammatory & metabolic & echocardiographic parameters across VASI tertiles (median and IQR based on 25^th^–75^th^ percentiles).

Parameter	Low VASI Median (IQR)	Medium VASI Median (IQR)	High VASI Median (IQR)	p-value
CRP (mg/L)	5.00 (1.09–13.01)	2.01 (0.56–4.15)	2.47 (1.99–3.55)	**0.030**
Total Cholesterol (mg/dL)	193 (159–221)	169 (161–212)	212 (169.5–221)	**0.020**
Non-HDL (mg/dL)	144 (109–161)	125 (116–162)	163 (133.5–171.5)	**0.003**
Triglycerides (mg/dL)	73 (56–113)	97 (66–173)	200 (122–207.5)	**<0.001**
HDL (mg/dL)	46 (39.9–60)	50 (39–52)	43 (39–53.5)	0.878
LDL (mg/dL)	113 (67.6–132.8)	103.8 (96.6–132.4)	130 (92.7–136.8)	0.422
VLDL (mg/dL)	14.6 (11.2–22.6)	19.4 (13.2–34.6)	40 (24.4–41.5)	**<0.001**
TG/HDL ratio	2.43 (1.09–2.65)	1.86 (1.27–3.97)	4.12 (2.18–5.44)	**<0.001**
AIP (log10 TG/HDL)	0.386 (0.037–0.424)	0.271 (0.103–0.598)	0.615 (0.338–0.735)	**<0.001**
Fibrinogen (mg/dL)	314 (269–402)	247 (219–333)	245 (223–307.5)	**0.001**
Aortic Root (mm)	28 (26–30.5)	30 (29.5–33)	30 (28.75–33)	**0.003**
Left Atrium (mm)	31 (28–36)	33 (31–35)	34 (32–35)	0.132
Ascending Aorta (mm)	31 (29–34)	31 (30–33.25)	31 (30–32)	0.287
Right Ventricle (mm)	32 (30–33)	32 (30–33)	32 (31–35)	**0.022**
LV Diastole (mm)	42 (41–45)	43 (42–45)	44 (42–47)	0.059
LV Systole (mm)	31 (29–33)	31 (31–33)	31 (31–33)	0.197
IVS (mm)	10 (8–10)	10 (9–10.5)	10 (10–10)	0.092
Aortic Gradient	5 (5–6.5)	6.5 (5–7)	7 (6–9.5)	**0.028**
Ejection Fraction (%)	60 (60–60)	60 (60–60)	60 (60–60)	0.602
EFT Parasternal (mm)	4 (2–4)	4 (2–4)	4 (3–7)	**0.045**
EFT Apical 4CH (mm)	3 (2.5–4)	3 (2–5)	4 (3–6)	**0.042**
EFT Subcostal (mm)	3 (2–4)	3 (2–4)	4 (3–5)	**0.002**
EFT Mean (mm)	3.0 (2.15–4.0)	3.3 (2.0–4.0)	4.0 (3.0–5.35)	**0.021**

CRP, C-Reactive Protein; HDL, High-Density Lipoprotein; LDL, Low-density lipoprotein; Non-HDL, Total cholesterol minus HDL; TG/HDL, Triglyceride-to-HDL ratio; AIP, Atherogenic Index of Plasma; IVS, Interventricular Septum; EF, Ejection Fraction; EFT, Epicardial Fat Thickness; PLAX, Parasternal Long Axis; A4C, Apical Four-Chamber view. Bold values indicate statistically significant p-values (p < 0.05).

Disease activity (active vs. stable) did not materially alter inflammatory, lipid, or EFT profiles; the only notable difference was a modest increase in aortic root diameter in active disease (33.5 vs. 30.0 mm, p = 0.020).

Subtype analyses revealed heterogeneity across phenotypes: acrofacial vitiligo (n = 25) showed higher CRP (p < 0.001), fibrinogen (p < 0.001), elevated non-HDL cholesterol (p = 0.010), and greater echocardiographic differences including larger aortic root (p = 0.001), left atrial diameter (p = 0.002), LV diastolic dimension (p = 0.003), IVS thickness (p = 0.023), and mean EFT (p = 0.040). Given the limited subgroup sizes, these findings should be considered hypothesis-generating.

Patients with higher BSA (≥10%) involvement exhibited significantly elevated triglycerides (p < 0.001), VLDL (p < 0.001), TG/HDL ratio (p < 0.001), and AIP (p < 0.001), along with increased EFT across parasternal (p = 0.026), apical four-chamber (p = 0.030), subcostal (p = 0.002), and mean (p = 0.016) measurements.

### Diagnostic performance for ıdentifying high VASI severity

**Figure 3:**
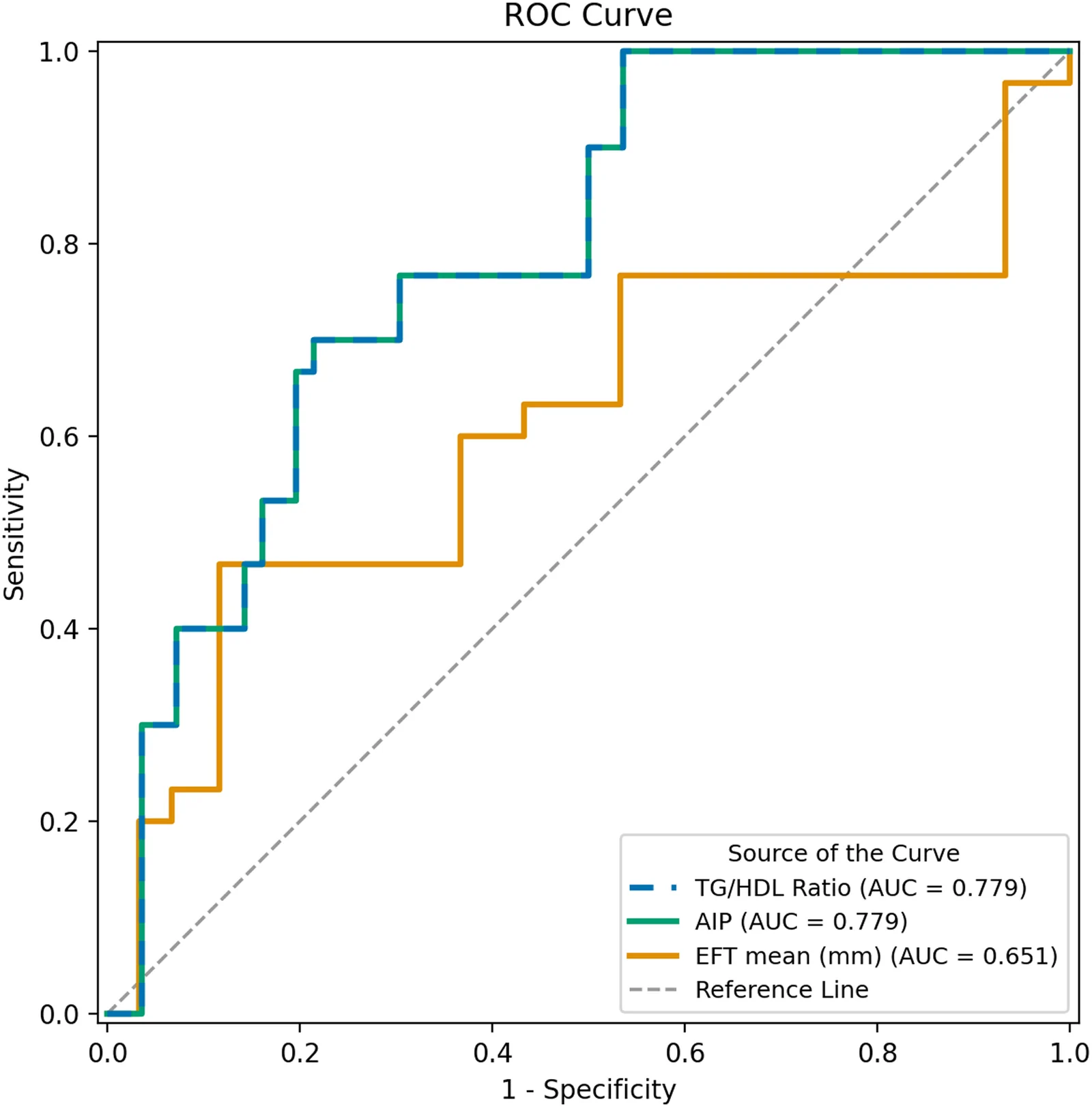
**Receiver Operating Characteristic (ROC) curves comparing the ability of lipid-based, and echocar diographic markers to discriminate high vitiligo severity (High VASI tertile).** Curves are shown for TG/HDL ratio, AIP, and mean Epicardial Fat Thickness (EFT). TG/HDL ratio and AIP demonstrated the highest discriminative performance for identifying high VASI severity. The TG/HDL ratio curve overlaps with the AIP curve (AUC = 0.779).

In ROC analysis (Fig. 3), TG/HDL ratio and AIP demonstrated strong performance for identifying high VASI severity (AUC = 0.779, p < 0.001). EFT demonstrated moderate predictive ability (AUC = 0.651, p = 0.025).

## Discussion

This study shows that vitiligo, even in the absence of overt cardiometabolic disease, is accompanied by measurable alterations in epicardial adiposity and selected cardiac structural parameters. Increased EFT across all imaging windows suggests that these differences are not explained by traditional cardiometabolic factors alone. Using multi-window EFT assessment alongside standardized severity measures allowed a more precise characterization of subclinical cardiovascular involvement. These findings align with evidence that vitiligo is associated with systemic metabolic and vascular abnormalities, including higher rates of metabolic syndrome, insulin resistance, dyslipidemia, and cardiovascular events.^4^, ^5^, ^6^, ^19^, ^20^

The consistent elevation of EFT in the vitiligo cohort despite exclusion of hypertension, diabetes, and dyslipidemia resembles observations in other inflammatory dermatoses where EFT correlates with systemic inflammation and oxidative stress.^9^, ^10^, ^11^, ^12^, ^13^, ^14^, ^15^ Evidence from carotid IMT studies likewise indicates that vitiligo may be accompanied by subclinical atherosclerotic changes despite the absence of overt comorbidities.^21^ Collectively, these observations suggest that vitiligo could be associated with early alterations in visceral cardiac adiposity comparable to those described in psoriasis and hidradenitis suppurativa.^10^, ^11^

As the present study population was restricted to exclude established cardiometabolic comorbidities, the true cardiovascular burden in vitiligo, particularly in patients with concurrent metabolic syndrome, dyslipidemia, or hypertension, may be more pronounced than the present findings suggest.

The associations between greater VASI/BSA involvement and higher triglyceride-related indices (triglycerides, VLDL, TG/HDL ratio, AIP), together with concurrent increases in EFT and aortic measurements, suggest that cutaneous disease burden may have systemic correlates. Prior work has shown that metabolic syndrome and subclinical atherosclerosis rise with increasing vitiligo severity, and these findings align with this pattern by demonstrating prominent alterations in triglyceride-rich lipoproteins even in metabolically “low-risk” individuals.^5^, ^21^

Phenotype-related differences further point to heterogeneity in systemic involvement. The acrofacialpattern displayed more adverse inflammatory and lipid profiles and greater echocardiographic differences. Literature indicates that acral involvement and non-segmental forms have stronger associations with insulin resistance, oxidative stress, and Koebner-related microtrauma, and acral involvement is associated with increased CRP and higher systolic blood pressure, which may help explain the more pronounced systemic signals observed in this subtype.^22^, ^23^, ^24^ Given the limited subgroup sample sizes, these observations should be considered hypothesis-generating.

The inverse associations between disease duration and several cardiometabolic measures, particularly the TG/HDL ratio and AIP, require careful interpretation. Cross-sectional sampling may underrepresent patients with long-standing disease who have developed comorbidities and were excluded by design; alternatively, systemic inflammatory–metabolic activity may be more prominent earlier in the disease course with attenuation over time.^21^, ^24^

Exploratory discrimination analyses showed that TG-based indices (TG/HDL ratio, AIP) distinguished patients with higher VASI severity. While not suitable for risk prediction, these lipid-derived measures may help identify individuals with a greater cardiometabolic burden, particularly when interpreted alongside echocardiographic findings.

This study has several limitations. Its cross-sectional design precludes any temporal or causal interpretation of the observed associations between vitiligo, cardiometabolic markers, and echocardiographic parameters. Although the strict exclusion of hypertension, diabetes, and dyslipidemia enhanced internal validity, this approach limits generalizability to broader vitiligo populations in whom such comorbidities are common. Thyroid disease was excluded based on clinical history; however, subclinical or undiagnosed thyroid dysfunction may have been present in some participants and cannot be ruled out as a residual confounder. Active disease was present in only a small subset of patients, reducing the power to detect activity-related differences. Subtype-specific analyses were also constrained by modest group sizes, and the heterogeneity observed across phenotypes should therefore be interpreted as exploratory. Although echocardiographic examiners were blinded to laboratory values, disease severity indices, and group allocation, complete diagnostic masking was not always achievable, as the presence of visible depigmentation may have allowed implicit identification of vitiligo cases in some participants. Interobserver reliability statistics were not calculated for echocardiographic measurements, which limits the quantitative assessment of measurement reproducibility. Cardiometabolic profiling was limited to laboratory indices and transthoracic echocardiography; more sensitive vascular assessments such as carotid intima-media thickness, pulse-wave velocity, flow-mediated dilation, or adipokine/cytokine panels were not performed. In addition, several potential confounders were not systematically assessed, including dietary habits, physical activity levels, smoking status, and socioeconomic status. These factors may independently influence lipid metabolism, systemic inflammation, and cardiovascular structure, potentially contributing to the observed associations beyond the direct effects of vitiligo. Although exclusion criteria were applied to minimize known metabolic confounding, residual lifestyle-related confounding cannot be fully excluded.

In conclusion, patients with vitiligo exhibited higher epicardial fat thickness and adverse TG-based atherogenic indices, together with subtle echocardiographic alterations, even in the absence of conventional cardiometabolic disease. The present findings indicate that vitiligo may encompass a measurable cardiometabolic component, particularly in patients with higher disease severity (VASI) and acrofacial or widely distributed phenotypes, and suggest that cardiovascular assessment may be considered in these subgroups, guided by future longitudinal evidence.

## ORCID IDs

Mustafa Esen: 0000-0002-4736-9142

Esin Diremsizoglu: 0000-0001-9824-481X

Murat Harman: 0000-0001-8444-4191

Kamran İldırımlı: 0000-0001-6608-871X

## Data availability statement

The data that support the findings of this study are available on request from the corresponding author.

## Ethics statement

All procedures adhered to the tenets of the Declaration of Helsinki and were approved by an Institutional Review Board (decision number: 2023/ 09-30).

## Research data availability

The entire dataset supporting the results of this study was published in this article.

## Financial support

None declared.

## Authors’ contributions

Mustafa Esen: Approval of the final version of the manuscript; data collection, analysis and interpretation; effective participation in research orientation; manuscript critical review; preparation and writing of the manuscript; statistical analysis; study conception and planning.

Abdullah Demirbaş: Approval of the final version of the manuscript; data collection, analysis and interpretation; effective participation in research orientation; manuscript critical review; preparation and writing of the manuscript; statistical analysis; study conception and planning.

Esin Diremsizoglu: Approval of the final version of the manuscript; data collection, analysis and interpretation; effective participation in research orientation; manuscript critical review; preparation and writing of the manuscript; statistical analysis; study conception and planning.

Murat Harman: Approval of the final version of the manuscript; data collection, analysis and interpretation; effective participation in research orientation; manuscript critical review; preparation and writing of the manuscript; statistical analysis; study conception and planning.

Kamran İldırımlı: Approval of the final version of the manuscript; data collection, analysis and interpretation; effective participation in research orientation; manuscript critical review; preparation and writing of the manuscript; statistical analysis; study conception and planning.

## Conflicts of interest

None declared.
